# Decapeptyl ameliorates cyclophosphamide-induced reproductive toxicity in male Balb/C mice: histomorphometric, stereologic and hormonal evidences

**Published:** 2013-10

**Authors:** Afsaneh Niakani, Farah Farrokhi, Shapour Hasanzadeh

**Affiliations:** 1 *Department of Biology, Faculty of Science, Urmia University, Urmia, Iran.*; 2 *Department of Basic Veterinary Science, Fculty of Veterinary Medicine, Urmia University, Urmia, Iran.*

**Keywords:** *Cyclophosphamide*, *Decapeptyl*, *Testis*, *FSH*, *LH*, *Testosterone*, *Mice.*

## Abstract

**Background:** Gonadotropin-releasing hormone (GnRH) is a reproductive key hormone. The GnRH analogues are widely used in in vitro fertilization and treatment of sex hormone-depended cancers induced by the materials used in chemotherapeutic agents.

**Objective: **The aim of this study is to evaluate the effects of cyclophosphamide and decapeptyl (analogues of GnRH) on histomorphometry and stereology of testicular tissue as well as gonadotropic and gonadal hormones indices in mice.

**Materials and Methods:** For this study, 24 adult male Balb/C strain mice were divided in four groups; first, cyclophosphamide (65 mg/kg/body weight (BW)), second, decapeptyl (0.05 mg/kg/BW), third, decapeptyl at first, and after 10 days of cyclophosphamide injection, and control group was received same volume of sterile saline. In order to evaluate the tissue changes in testes of the mice, sections were prepared and stained with Hematoxylin-Eosine, Periodic Acid Schief's (PAS) and Oil-Red-O staining techniques.

**Results: **The cyclophosphamide causes histomorphologic changes in the testicular tissue; whereas such changes by decapeptyl were comparatively mild. The morphometric results revealed significant reduction in diameters of seminiferous tubules (p=0.02), and the stereological results confirmed significant differences in spermatogenesis (SI) as well as rate of tubal differentiation (TDI) indices between experimental and control groups (p=0.001). In addition, the morphometric findings proved that, there are significant decrease (p=0.001) in thicknesses of epithelia and stereologic result revealed reduction in number of cell layers in both decapeptyl and chemotherapy groups, but the decrements of these parameters were significant (p=0.02) in later group. In groups that had received cyclophosphamide, and decapeptyl alone, the LH and testosterone levels were decreased significantly (p=0.03), whereas in those that had received decapeptyl along with cyclophosphamide, the LH and FSH levels showed a decline but the level of testosterone increased.

**Conclusion:** These results demonstrated that, analogue of GnRH i.e., decapeptyl protect morphologic, morphometric, and stereologic alterations of the testes tissue, as well as gonadotropic and gonadal hormonal changes preceding cyclophosphamide treatment in male mice.

This article extracted from M.Sc. thesis. (Afsaneh Niakani)

## Introduction

The gonadotropic releasing hormone (GnRH) plays a vital role in the control of reproduction by stimulating the release of pituitary luteinizing hormone (LH) and follicle-stimulating hormone (FSH), (1). There are differences in responsiveness of hypothalamus to low intermittent and continuous chronic stimulus by GnRH or GnRH agonist (2-4). The desensitization of gonadotrope cells involves a down-regulation of pituitary receptors for GnRH by mechanisms, some of which still remained to be clarified (5, 6). According to prior reports, the agonistic analogous of GnRH such as decapeptyl have gained clinical indications (7-9). 

Storehouse formulations of GnRH antagonists such as cetrorelix and cyclophosphamide could become an important addition to the clinical armamentarium. Infertility represents one of the main long-term consequences of combination chemotherapy given for cancer disease, and other malignancies (10, 11). Since dividing cells are known to be more sensitive to the cytotoxic effects of alkylating agents than are cells at rest, it has been suggested that, inhibition of the pituitary-gonadal axis would reduce the rates of spermatogenesis as well as oogenesis and thereby render the germinal epithelium less susceptible to the effects of chemotherapy (12, 13). 

The possibility of administering an adjuvant treatment that might limit the gonadal damage caused by, and otherwise successful treatment programme is therefore attractive (14). Glode *et al *tested this hypothesis using a murine model and concluded that an agonistic analogue of GnRH appeared to protect male mice from the gonadal damage normally produced by cyclophosphamide (15). The decreased secretion of the pituitary gonadotropines, along with decreasing gonadal function, could protect against the sterilizing effects of chemotherapy. Spermatogenesis occurs within the seminiferous tubules of the testes, and during this process, male germ cells progress through three distinct phases. 

According to a recent study, the cyclophosphamide causes significant decreases in the body, testes and epididymides weights as well as many histological alterations, spermatogenic activities and testicular antioxidant capacity along with epididymal sperm count and serum testosterone concentration in adult male rats (16). More detail studies have been done on the effects of GnRH and its analogues on the protection of ovaries against damages of cytotoxic chemotherapy like cyclophosphamide in female mice and rats, but in male mice, less is known about the possibility for testicular protection through endocrine manipulation. 

Based on the pervious findings, the aim of this study was to investigate and compare the effects of GnRH agonist i.e., decapeptyl and antagonist i.e., cyclophosphamide on the reproductive system in mouse model.

## Materials and methods

For this experimental study, 24 adult and apparently healthy male Balb C/ mice with average weight of 20.00±3.00 gr were obtained from animal house of Veterinary School of Urmia University. They were housed in a specific pathogen-free environment under standard conditions of temperature (25±2^o^C), relative humidity (50±10%) and light (12h light/dark). They were fed with a standard pellet diet and had free access to water. 

Clinical and behavioral observations were also recorded throughout the study. Animal work was conducted in compliance with guidelines for the human care and use of laboratory animals using protocols approved by the university. Theluteinizing hormone releasing hormone (LHRH) agonist (Decapeptyl) was extended by phosphate buffer saline (PBS). A depot formulation of the LHRH antagonist vial containing the 200mg powder of cyclophosphamide which is solved in 100 ml of distilled water. After adding saline (Distilled water), the vial was given severely shakes till the powder is completely resolved and a clear, colorless solution is formed. 

Group 1 was received a single cyclophosphamide (65 mg/kg BW) injection through intraperitoneal route. Group 2 was received a single dose of Decapeptyl (0.05 mg/kg BW) through intraperitoneal route. Group 3 was received an intraperitoneal injection of decapeptyl (0.05 mg/kg BW) at first, and 10 days after be given single dose of cyclophosphamide (65 mg/kg BW). Group 4 was received an intraperitoneal injection of the vehicle (sterile distilled water). 30 days after commence of experiment, all animals in 4 groups at first were anesthetized by Ether Vapor, and then their blood collected through intracardial route. Then immediately the serum of the samples were separated and put in deep freeze for further investigations. In the second step, after expire of all mice, their abdominal region were cut opened and their testes taken out and put in Bouin’s solution for fixation. 

**Histologic and histomorphometric experiments**

For these purposes the fixed testes were processed through routine paraffin embedding technique, cut at 7µm thicknesses and stained by hematoxylin-eosin procedure. By the use of morphometric lens device (Dino lite Capture 1, Dino Lite Co. French), all the morphometric parameters including; diameters and epithelial thicknesses of semniferous tubules (SFTs), were recorded. The diameters of seminiferous tubule were measured by using Soudamani *et al* formula (17). Tubules in each of 25 randomly selected cross-sections of testes and the mean tubular diameter, with large and small diameter of each tubule measured using a calibrated micrometer lens device connected to the optical microscope and were calculated by using the following formula.

$\mathrm{mean diameter}=\sqrt[{2}]{\mathrm{magnification}\times B\times L}$

L= length (large diameter) and B= width (small diameter). For spermatogenesis index (SI), transect of each 100 fields in each group were studied with light microscopy. The number of tubules containing spermatozoa and spermatids were counted. For the calculation of tubule differentiation index (TDI), 200 cross-sections of seminiferous tubules were randomly analyzed in each mouse (one hundred per testis). TDI is the percentage of seminiferous tubules containing at least four differentiated germ cells (18).

The average number was calculated for each group according to Meistrich *et al* procedure (19). Results are expressed as Mean±SEM. For the statistical analyses of data the computer software SIGMASTAT was used. In results were subjected to one-way ANOVA, followed by Bonferroni t*-*test, and p<0.05 was accepted as statistically significant.

**Histochemical experiments**

**Periodic Acid Schiff (PAS)**: for the identification and qualification of polysaccharides and inert mucous compounds in testicular tissues, the formalin fixed and paraffin embedding technique was used (20).

**Oil-Red-O**: For this purpose, frozen sections were prepared by cryostat. The most of exogenous and endogenous lipid compounds such as phospholipids, saturated lipids and sterols are identifiable by this method (21). For the recognition of the steroids in the leydig's cells the Oil-Red-O technique was adopted by frozen sectioning (22).

**The hormonal assay**s**:** The assays of LH and FSH (Pishtaz Teb kits, Zaman Diagnostics Tehran, Iran), were carried out on blood serum by using an ELISA technique, and testosterone assay were carried out on blood serum by ELISA kit(Testosterone rat/mouse ELISA kit, Cat.^-^No.: DEV9911, Demeditec Diagnostics GmbH, Germany). For the LH intra-assay and inter-assay, if frequency of repetition was 24, the amount of SD (in international units per liter) was 0.53 and 0.45, respectively. For FSH intra-assay and inter-assay, if the frequency of repetition was 24, the amount of SD was 0.14 and 0.45 IU/L, respectively.

**Statistical analysis**

Results are expressed as Mean±SE. Differences between groups were assessed by the analysis of variance (ANOVA) using the SPSS software package for Windows. Statistical significance between groups was determined by Tukey multiple comparison post hoc test and the p<0.05 were considered to be statistically significant.

## Results

**Histomorphological findings**

**Figure 1:**
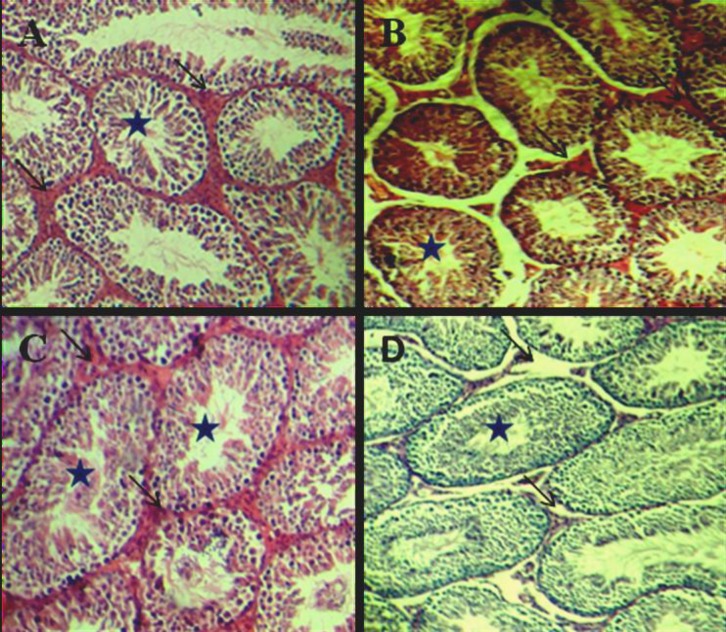
A: cross section from testis of the mice belonging to the control. B: cyclophosphamide treated C: decapeptyl treated D: the cyclophosphamide +decapeptyl treated groups. H& E. X100

In the control group, all histomorphological features in the seminiferous tubules (SFTs) were normal and no abnormality was observed (Figure 1-A). In test group 1, which received cyclophosphamide at rate of 65 mg/kg BW, the degenerative and destructive sings were higher than other groups (Figure 1-B). In test group 2, which received decapeptyl at rate of 0.05 mg/kg BW, no apparent changes in the seminiferous tubules and/or in spermatogonia were observed under light microscopy. Accumulations and clumping of the spermatozoa were also seen in the center of the STs (Figure1-C). 

**Figure 2:**
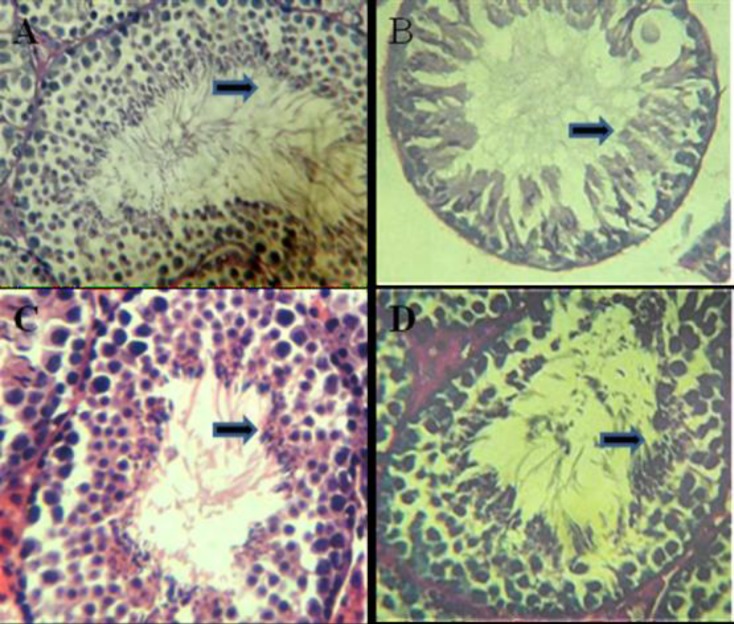
A: cross section from testis of the mice belonging to the control, B; cyclophosphamide treated C: decapeptyl treated D: the cyclophosphamide + decapeptyl treated groups.PAS. X400

In test group 3, which received cyclophosphsmide+ decapeptyl, the degenerative and destructive sings were declined in comparison to test group 1 (Figure 1-D). In the control group, all of histomorphological features in the seminiferous tubules (SFTs) including spermatogonia types A and B, spermatocytes type I, spermatids and spermatozoa were normal and no abnormality was observed (Figure 2-A). 

In test group 1, which received cyclophosphamide at rate of 65 mg/kg BW, the degenerative and destructive sings were higher than other groups and the population of all cell types, including spermatogonia types A and B, spermatocytes, spermatids and spermatozoa were significantly reduced (Figure 2-B). In test group 2, which received decapeptyl at rate of 0/05mg/kg BW, no apparent changes in the cell populations of seminiferous tubules were seen (Figure 2-C). 

**Figure 3:**
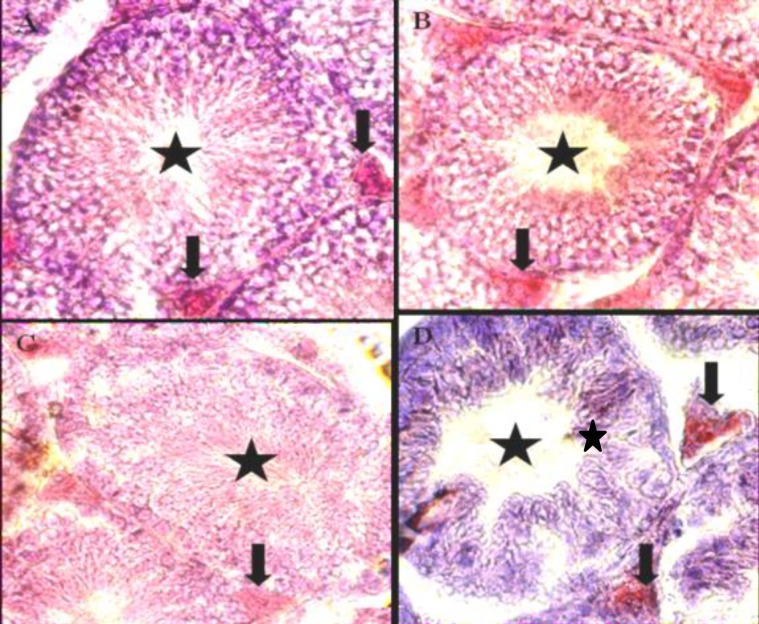
A: cross section from testis of the mice belonging to the control , B; cyclophosphamide treated C: decapeptyl treated D: the cyclophosphamide + decapeptyl treated groups. The Semniferoustubles ( ), and Leydigs' Cells ( )Oil Red O X250

In test group 3, which received cyclophosphsmide + decapeptyl, the degenerative and destructive sings were declined in comparison to test group 1, however here a little histological changes were present (Figure 2-D). In control group, all histomorphological features in the STs and leydig's cells were normal (Figure 3-A). In test group 1, which received cyclophosphamide at rate of 65 mg/kg BW, the degenerative and destructive sings were seen in spermiogenic lineage as well as interstitial cells (Leydig´s cells) (Figure 3-B). 

In test group 2, which received decapeptyl at rate of 0/05mg/kg BW, no evident changes in the seminiferous tubules and leydig's cells were seen (Figure 3-C). In test group 3, which received cyclophosphsmide+ decapeptyl, the degenerative and destructive sings were declined in comparison to group 1 (Figure 3-D).

**Morphometric findings**

**Figure 4:**
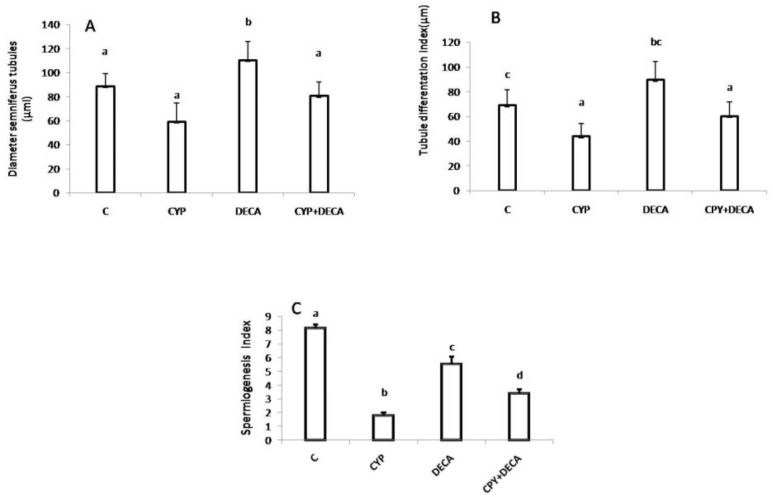
A: comparative representation of the mean diameters of somniferous tubules, B: the tubule diffirentation index (TDI) of the somniferous tubules, C: Spermatogenesis index (SI) in controls and test groups

There were significant decrease (p=001) in thicknesses of epithelia in both decapeptyl and chemotherapy groups, but the decrements of these parameters were significant (p=0.02) in later group. The group which received cyclophosphamide, were showing significantly (p=0.02) decrease in the mean diameter of SFTs in comparison to the controls. Meanwhile, in decapeptyl treated group sharp increase in this parameter was evident (Figure 4-A). 

The group which received cyclophosphamide, were showed significantly (p=0.001) decrease in TDI in comparison to the controls. Meanwhile, in decapeptyl treated group sharp increase in this parameter was evident, and in cyclophosphamide+ decapeptyl group the restoration of this parameter was reached to the level of controls (Figure 4-B). In all of test groups, the spermatogenesis index (SI) was significantly (p=0.001) declined in comparison to the controls (Figure 4-C).

**Hormonal assays**

**Figure 5:**
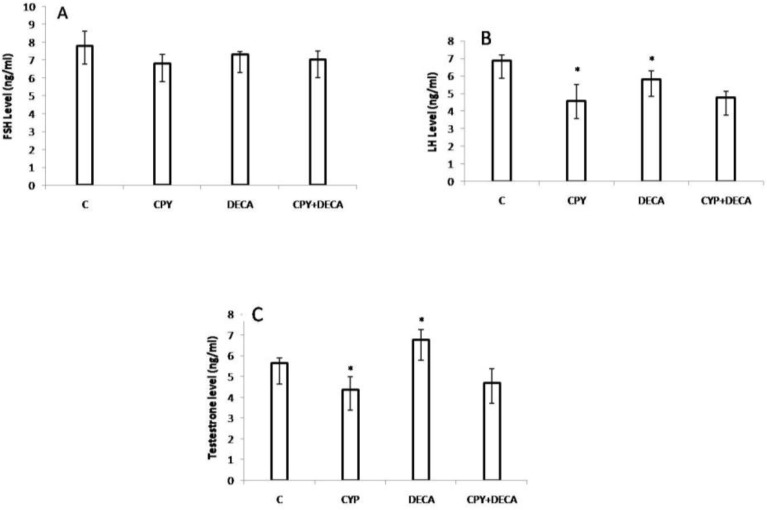
A: the FSH levels, B: the LH levels C: the testosterone levels in control and different test groups

There was not significant (p>0.05) alterations in the levels of FSH in control and different test groups (Figure 5-A). There was significant (p=.03) decrease in the levels of LH in cyclophosphamide and cyclophosphamide+ decapeptyl groups (Figure 5-B). 

There was significant (p=0.03) decrease in the level of testosterone in group which received cyclophosphamide, whereas there was significant (p=0.03) increase in the level of this hormone in decapeptyl group in comparison to the all other groups (Figure 5-C).

## Discussion

For assessment and confirmation of the cyclophosphamide and decapeptyl (analogues of GnRH) effects on the stereology of testicular tissue as well as gonadotropic and gonadal hormones indices in mice, this study was planned and performed. The results confirmed that, cyclophosphamide causes histomorphologic changes in the testicular tissue; whereas by the uses of decapeptyl, the changes were comparatively milder. 

The morphometric as well as stereological results were revealed significant reduction in diameters of seminiferous tubules, spermatogenesis (SI) as well as rate of tubal differentiation (TDI) indices in experimental groups, but these reductions were enhanced in group which received cyclophosphamide alone. The reductions in the levels of the FSH in test groups in comparison to controls were not significant, whereas, the levels of LH, in all of test groups particularly, in those received the cyclophosphamide alone, were highly reduced. The level of testosterone in group which received the cyclophosphamide alone was highly reduced, but in test group which received the decapeptyl alone, was highly increased. The level of testosterone in group which received cyclophosphamide plus decapeptyl almost increased to level in control group.

According to Meistrich *et al* the chemotherapy affecting the spermatogenic functions of testicular tissue, and the fast dividing cells in this tissue including spermatogonia are more susceptible to this cytotoxic possessions (23). As declared by Baker *et al* in the majority of the patients with testicular germ cells tumor, the sperm count is reduced (24). The germinal epithelium of the adult testis is more susceptible to damage than that of the prepubertal testis, and in contrast to naturally occurring GnRH, the GnRH agonists, after producing an initial stimulation of gonadotropin release for approximately 2 weeks, lead to GnRH receptor down-regulation and thereby to supress of gonadotropins and sex hormones (25). 

According to report of Waxman *et al* in contrast, GnRH antagonists cause competitive blockage of pituitary GnRH receptors and lead to an immediate and effective suppression of LH, FSH, and gonadal hormones (26). Cyclophosphamide affects the rapidly proliferating cells in the seminiferous tubules due to its cytotoxic property, and would hypothetically reduce the number of spermatozoa that would be produced when the testes become functional. Cyclophosphamide when given at a low dose for only 1 week, produces an increase in post-implantation loss, suggesting that the drug may affect spermatozoa after they have left the testis while they are maturing in the epididymis (27). 

According to the results of previous reports, the dividing cells are more sensitive to the cytotoxic effects of alkylating agents than the cells at rest, and it has been suggested that inhibition of the pituitary-gonadal axis would reduce the rate of spermatogenesis as well as oogenesis, thus rendering the germinal epithelium less susceptible to the effects of chemotherapy (12, 13). 

According to Waxman *et al* supplementation of chemotherapeutic agent by an adjuvant could limit the gonadal damages after treatment programme (26). Glode *et al* experienced this suggestion using a murine model and concluded that an agonistic analogue of GnRH appeared to protect male mice from the gonadal damage normally produced by cyclophosphamide (15). 

The results of this investigation revealed that, the cyclophosphamide reduces the population of germ cells linage including spermatogonia, spermatocytes, spermatids as well as spermatozoa. Peirouvi *et al* reported that there is no significant difference in diameters of tubules between experimental (were received GnRH agonist) and control groups, but significant difference in lumen diameter, thickness of epithelium and the number of cell layers in seminiferrous tubules are obvious (27, 28). Nseyo *et al* reported a recovery of spermatogenesis in a dog pretreated with the GnRH agonist i.e., buserelin, 6 months after exposure to this agent (29). 

Our results revealed that, treatment with decapeptyl returns the microarchitecture and cellularity of SFTs to almost normal level. According to a report, buserelin failed to preserve fertility in men and women treated with cytotoxic treatment with their cyclophosphamide for Hodgkin’s disease (26). The results of our study revealed that is difference in the spermiogenesis index, tubule differentiation index and seminiferous tubular diameter after exposure to cyclophospghamide. Satoh *et al* also reported the same results (30).

In a normal status, secretion of GnRH from hypothalamus stimulating the anterior pituitary gland to produce FSH and LH which in turn bring about gonadal hormones secretions (testosterone and estrogen) in testes. Reduced hypothalamic sensitivity to negative feedback effects of androgens increases GnRH secretion and consequently the amount of gonadotropin and androgen secretion increases which cause testicular growth and the incidence of other secondary sex characteristics (8). Decapeptyl which is one of the synthetic GnRH analogues which is used in treatment and control of reproductive system disorders in males and females. 

GnRH analogues with continuous stimulation of GnRH secretion, inhibits the decrement in secretion of LH and FSH hormones (31). A GnRH analogue increases the secretion of LH and FSH in first injection, but thereafter, despite the presence of GnRH, gonadotropin secretion decreases (32). Gonadotropin receptors are located on the testis and seminiferous tubules, decreased spermatogenesis which is caused by the chemotherapic agent disrupts the pituitary gonadal axis. In the mice chemotherapy the level of FSH is controlled based on spermiogenesis. Because the inhibition of meiosis by the chemotherapic agent, the spermiogenesis phase is quite impossible to observe (33, 34). In the present study the levels of FSH and more distinctly LH are reduced, consequently the spermatogenesis negatively affected by chemotherapy. According to Brinkworth cyclophosphamide-induced genetic damage to cells causes sexual harm (35). 

According to Masta *et al* cross connections in the active parts of chromosomes are damage in chemotherapy (36). Evidences show that, the damages to the DNA of the male sex cells caused by chemicals and drugs lead to gene mutations and consequently congenital malformation which are transferable (35). Men treated with anticancer drugs are more likely to experience permanent infertility and defects in the gonads (37). Since the male sex cell division rate is very high, thus it is sensitive to anti-cancer or antimitotic agents. The results of this study confirmed that tissue damages in testicular tissue are higher in cyclophosphamide treated group than in the group treated with cyclophosphamide plus decapeptyl and, so the diameter of the seminiferous tubes in the group treated with cyclophosphamide was reduced whereas, the diameter of this tubules was significantly increased in decapeptyl treated group. One can conclude that the decapeptyl has protective effect on seminiferous tubules damages in consequence to cyclophosphamide treatment. These findings are consistent with results of the previous reports (38, 39).

In most of the cases, azoospermia and oligospermia are consequents of cancer therapy in males. According to Howell and Shalet, the germinal epithelium in the testis is more vulnerable to chemotherapy than those of mature adult cells (25). The results of this study were revealed that, the differentiation factor for testicular seminiferous tubules in the group treated with cyclophosphamide significantly was reduced. The loss was more enhanced in cyclophosphamide compared with a decapeptyl, and control groups, whereas the group that has received decapeptyl along with cyclophosphamide, the damage was milder.

The damages caused by chemotherapy or radiation therapy could be on somatic cells of the testis, like as Sertoli and leydig’s cells (40). Our results on the investigation of the leydig's cells were revealed that in the cyclophosphamide group the destruction of these cells are taking place. The abnormal testosterone levels following chemotherapy are due to the leydig's cells damages which is a side effect outcome of the chemotherapy (41-43). Since the testosterone is a key hormone for proper function of the accessory sex glands in the male mice, thus destructive effects of chemotherapic agent, here cyclophosphamide, on these glands, will negatively directs the function of these gland and thus would reduce in reproductive potentials. 

According to previous reports (41, 43) and results of this study decapeptyl has restorative and ameliorative effects on the destructive outcomes of the chemotherapy. In this study, a significant reduction in serum testosterone levels in the group treated with cyclophosphamide was observed, which is a clear evidence of toxic effects of this drug on the leydig's cells. 

The present study showed that, chemotherapy with cyclophosphamide in male mice cause changes in the testes histology and treatment with decapeptyl brings about protection of seminiferous tubules.

## Conflict of interest

The authors declare that there are no conflicts of interest in this study.

## References

[1] Clayton R (1989). Gonadotrophin-releasing hormone: its actions and receptors. J Endocrinol.

[2] Shupnik MA (1990). Effects of gonadotropin-releasing hormone on rat gonadotropin gene transcription in vitro: requirement for pulsatile administration for luteinizing hormone-beta gene stimulation. Mol Endocrinol.

[3] Haisenleder DJ, Ortolano GA, Yasin M, Dalkin AC, Marshall JC (1993). Regulation of gonadotropin subunit messenger ribonucleic acid expression by gonadotropin-releasing hormone pulse amplitude in vitro. Endocrinology.

[4] Schally AV (1999). Luteinizing hormone-releasing hormone analogs: their impact on the control of tumorigenesis. Peptides.

[5] Schally AV, Comaru-Schally AM, Gonzalez-Barcena D, Reissmann T, Engel J, Minaguchi H, Sugimoto O (1997). Endometriosis Today: Advances in Research and Practice.

[6] Schally AV, Comaru-Schally AM, Holland JF, Frei E, Bast RC, Kufe DE, Pollock RE, Weichselbaum RR (2000). Cancer Medicine.

[7] Schally AV (1999). Gynecol LH-RH analogues: I. Their impact on reproductive medicine. GynecolEndocrinol.

[8] Cook T, Sheridan WP (2000). Development of GnRH antagonists for prostate cancer: new approaches to treatment. Oncologist.

[9] Schally AV, Halmos G, Rekasi Z, Arencibia JM, Devroey P (2001). Infertility and Reproductive Medicine Clinics of North America.

[10] Muller U, Stahel RA (1993). Gonadal function after MACOP-B or VACOP-B with or without dose intensification and ABMT in young patients with aggressive non-Hodgkin's lymphoma. Ann Oncol.

[11] Glaser SL (1994). Reproductive factors in Hodgkin's disease in women a review Am. J Epidemiol.

[12] Johnson DH, Linde R, Hainsworth JD, Vale W, Rivier J, Stein R (1985). Effect of a luteiruzing hormone releasing hormone agonist given dunng combination chemotherapy on post-therapy fertility in male patients with lymphoma preliminary observations. Blood.

[13] ShennsRJ, DeVita VT Jr, Hellman S, Rosenberg SA (1993). Gonadal dysfunction. Cancer- Principles and Practice of Oncology.

[14] Wheeler GP (1962). Studies related to the mechanism of action of cytotoxic alkylating agents. Cancer Res.

[15] Glode LM, Robinson J, Gould SF (1981). Protection from cyclophosphamide induced testicular damage with an analogue of gonadotrophin-releasing hormone. Lancet.

[16] Shalizar Jalali A, Hasanzadeh Sh, Malekinejad H (2012). Achillea millefoliumin florescence aqueous extract ameliorates cyclophosphamide-induced toxicity in rat testis: stereological evidences. Chin J Nat Med.

[17] Soudamani S, Yuvaraj S, Malini T, Balasubramanian K (2005). Exprimental diabetes has advers effects on the differentiation of ventral prostate during sexual maturation of rats. Anat Rec DiscovMol Cell Evol Biol.

[18] Porter KL, Shetty G, Meistrich ML (2006). Testicular dema is associated with spermatogonial arrest in irradiated rats. Endocrinol.

[19] Meistrich ML, Wilson G, Porter KL, Huhtaniemi I, Shetty G, Shuttlesworth GA (2003). Restoration of spermatogenesis in dibromochloropropane (DBCP)-treated rats by hormone suppression. Toxicol Sci.

[20] Lee G, Luna HT (1968). Manual of Histological Staining Methods of the Armed Forces Institute of Pathology.

[21] Collins K, Geisinger K, Wagner P, Blackburn K, Washburn L, Block S (1995). The cytologic evaluation of lipid-laden alveolar macrophages as an indicator of aspiration pneumonia in young children. Arch Pathol Lab Med.

[22] Gretchen L ( 1979). Animal Tissue Techniques.

[23] Meistrich ML (1986). Relationship between spermatogonial stem cell survival and testis function after cytotoxic therapy. Br J Cancer Suppl.

[24] Baker JA, Buck GM, Vena JE, Moysich KB (2005). Fertility patterns prior to testicular cancer diagnosis. Cancer Causes Control.

[25] Howell S, Shalet S (1998). Gonadal damage from chemotherapy and radiotherapy. Endocrinol Metab Clin North Am.

[26] Waxman JH, Ahmed R, Smith D, Wrigley PF, Gregory W, Shalet S (1987). Failure to preserve fertility m patients with Hodgkin's disease. Cancer Chemother Pharmacol.

[27] Vickery BH (1986). Comparison of the potential for therapeutic utilities with gonadotropin releasing hormone agonists and antagonists. Endocrin Rev.

[28] Peirouvi T, Farjah G, Soleimani Rad J, Ghaffari Novin M (2007). Vitrification induced apopto-sis in spermatozoa from fertile and subfertile men. Iran J Reprod Med.

[29] Nseyo UO, Huben RP, Klioze SS, Pontes JE (1985). Protection of germinal epithelium with luteinizing hormone-releasing hormone analogue. J Urol.

[30] Satoh K, Ohyama K, Nakagomi Y, Ohta M, Shimura Y, Sano T (2002). Effects of growth hormone on testicular dysfunction induced by cyclophosphamide (CP) in GH-deficient rats. Endocrinol J.

[31] Hellerstedt BA, Pienta KJ (2002). The current state of hormonal therapy for prostate cancer. CA Cancer J Clin.

[32] Brawer MK (2001). The evolution of hormonal therapy for prostatic carcinoma. Rev Urol.

[33] Tohda A, Matsumiya K, Tadokoro Y, Yomogida K, Miyagawa Y, Dohmae K (2001). Testosterone suppresses spermatogenesis in juvenile spermatogonial depletion (jsd) mice. Biol Reprod.

[34] Shetty G, Weng CC, Meachem SJ, Bolden-Tiller OU, Zhang Z, Pakarinen P (2006). Both testosterone and FSH independently inhibit spermatogonial differentiation in irradiated rats. Endocrinology.

[35] Brinkworth MH (2000). Paternal transmission of genetic damage: findinds in animals and humans. Int J Androl.

[36] Masta A, Gray PJ, Philips DR (1994). Molecular basis of nitrogen mustard effects on transcription process: role of depurination. Nucleic Acids Res.

[37] Kenney LB, Laufer MR, Grant FD, Grier H, diller L (2001). High risk of infertility and long term gonadal damage in males treated with high does cyclophosphamide for sacoma during childhood. Cancer.

[38] Roeser HP, Stocks AE, Smith AJ (1978). Testicular damage due to cytotoxic drugs and recovery after cessation of therapy. Aust NZ J Med.

[39] Schilsky RL, Lewis BJ, Sherins RJ, Young RC (1980). Gonadal dysfunction in patients receiving chemotherapy for cancer. Ann Intern Med.

[40] Meistrich ML, Finch M, da Cunha MF, Hacker U, Au WW (1982). Damaging effects of fourteen chemotherapeutic drugs on mouse testis cells. Cancer Res.

[41] Klingmuller D, Schepke M, Enzweiler C, Bidlingmaier F (1993). Hormonal responses to the new potent GnRH antagonist Cetrorelix. Acta Endocrinol.

[42] Thomson AB, Campbell AJ, Irvine DC, Anderson RA, Kelnar CJ, Wallace WH (2002). Semen quality and spermatozoal DNA integrity in survivors of childhood cancer: a case-control study. Lancet.

[43] Klein EC (2008). Gonadal complications, Section 40. Complications of cancer and its treatment. Cancer medicine.

